# Comparison of solid and liquid fractions of pretreated Norway spruce as reductants in LPMO-supported saccharification of cellulose

**DOI:** 10.3389/fbioe.2022.1071159

**Published:** 2022-12-13

**Authors:** Chaojun Tang, Madhavi Latha Gandla, Leif J. Jönsson

**Affiliations:** Department of Chemistry, Umeå University, Umeå, Sweden

**Keywords:** lignocellulose bioconversion, Norway spruce, enzymatic saccharification, lytic polysaccharide monooxygenase, cellulose, reductant, lignin, pretreatment liquid

## Abstract

The role of lignin in enzymatic saccharification of cellulose involving lytic polysaccharide monooxygenase (LPMO) was investigated in experiments with the solid and liquid fractions of pretreated Norway spruce from a biorefinery demonstration plant using hydrothermal pretreatment and impregnation with sulfur dioxide. Pretreated biomass before and after enzymatic saccharification was characterized using HPAEC, HPLC, *Py*-GC/MS, 2D-HSQC NMR, FTIR, and SEM. Chemical characterization indicated that relatively harsh pretreatment conditions resulted in that the solid phase contained no or very little hemicellulose but considerable amounts of pseudo-lignin, and that the liquid phase contained a relatively high concentration (∼5 g/L) of lignin-derived phenolics. As judged from reactions continuously supplied with either air or nitrogen gas, lignin and lignin fragments from both the solid and the liquid phases efficiently served as reductants in LPMO-supported saccharification. When air was used to promote LPMO activity, the enzymatic conversion of cellulose after 72 h was 25% higher in reactions with pretreated solids and buffer, and 14% higher in reactions with pretreatment liquid and microcrystalline cellulose. Research in this area is useful for designing efficient saccharification steps in biochemical conversion of lignocellulosic biomass.

## 1 Introduction

Lignocellulose is an abundant bioresource that has gained wide attention in the production of bio-based commodities (Zhang, 2008). Enzymatic deconstruction of cellulose and hemicelluloses in lignocellulosic biomass to sugars offers a renewable alternative to the refining of fossil resources, which are associated with environmental problems and problems concerning energy security (Kaparaju et al., 2009). Biochemical conversion typically includes hydrothermal pretreatment, enzymatic saccharification of cellulose, microbial fermentation of sugars, and valorization of the lignin-rich solid residue, often referred to as hydrolysis lignin (Martín et al., 2022). The challenges of biochemical conversion are highly dependent on achieving high sugar yields from cellulose at low cost, thus making it more competitive (Østby et al., 2020).

Enzymatic saccharification of cellulose has typically been achieved using hydrolytic enzymes, such as cellobiohydrolase, endoglucanase, and β-glucosidase (Van Dyk and Pletschke, 2012). More recently, an oxidoreductase called LPMO (lytic polysaccharide monooxygenase) has emerged as a new tool in the deconstruction of recalcitrant lignocellulose, contributing to higher sugar yields (Horn et al., 2012). LPMO is a typical component of currently available commercial enzyme cocktails due to its ability to boost the action of enzymes involved in saccharification of cellulose. It serves as a complement to hydrolytic enzymes due to its ability to catalyze oxidative cleavage of glycosidic bonds of cellulose to create more targets for hydrolysis, which is achieved in the presence of molecular oxygen as co-substrate and an electron donor (Hemsworth et al., 2015).

LPMO-catalyzed reactions can involve different types of electron donors, both small and large molecules. Small organic molecules that serve as electron donors include ascorbic acid (Vaaje-Kolstad et al., 2010) and gallic acid (Quinlan et al., 2011). Larger molecules, such as fungal CDH (cellobiose dehydrogenase) (Phillips et al., 2011), polyphenol oxidase (Frommhagen et al., 2017), and glucose-methanol-choline (GMC) oxidoreductase, have also been proposed to serve as electron donors (Kracher et al., 2016).

Lignin, one of the main components of lignocellulosic biomass, may also increase the activity of GH61 enzymes (later renamed as auxiliary activity family AA9, LPMO) (Dimarogona et al., 2012). Lignin has been proposed to be an electron donor since LPMO has been found to have the ability to oxidize lignocellulosic substrates without addition of an external electron donor, such as ascorbic acid (Cannella et al., 2012; Westereng et al., 2015). This is industrially important, because if lignin or lignin derivatives are sufficient to sustain LPMO-supported saccharification, addition of costly external reaction chemicals, such as ascorbic acid, can be minimized. However, relatively few studies have so far focused on reactions with real industrial lignocellulosic substrates, commercial cellulase cocktails under industrially relevant conditions, and with a controlled supply of gas in the reaction mixtures.

Other reasons for studying lignin and lignin derivatives as electron donors for LPMO include that the lignin content and type of lignin of lignocellulosic biomass differ depending on the biological origin, and that the structure and the fraction of lignin in the raw material are affected in different ways by different types of pretreatments and pretreatment conditions (Martín et al., 2022). The lignin content (DW, dry-weight) in biomass differs from below 20% in grasses and many agricultural residues (Ralph et al., 2004) to around 30% in softwood (Sjöström, 1993). Furthermore, the phenylpropane units in softwood lignin are predominantly G (guaiacyl) units, whereas the lignin of many agricultural residues consists of considerable proportions of G, S (syringyl), and H (*p*-hydroxyphenyl) units (Ralph et al., 2004). The fractions of different interunit linkages also vary in different forms of biomass, although the β-O-4-aryl ether linkage is the most common (Ralph et al., 2004). Although it is hemicellulose rather than lignin that is the target of hydrothermal pretreatment under acidic conditions (Galbe and Wallberg, 2019; Martín et al., 2022), the lignin is also affected. A minor fraction of the lignin is degraded and ends up as phenylic substances (i.e., phenolic and non-phenolic aromatic substances) in the liquid phase after the pretreatment (Ko et al., 2015; Jönsson and Martín, 2016). So far, the role of lignin-derived phenolics as inhibitors has received considerable attention (Jönsson and Martín, 2016), whereas their potential positive role as electron donors for LPMO is not well understood. Due to the large differences between different lignins, more studies on the action of LPMO are needed, especially with lignins and lignin derivatives from industrially important feedstocks and pretreatment processes.

Depending on the industrial process scenario, the liquid phase of pretreated biomass (i.e., the pretreatment liquid or the hemicellulosic hydrolysate) could either be included or excluded during enzymatic saccharification. A common approach is to use a slurry (i.e., a mixture of the solid and the liquid phases obtained after pretreatment) as a substrate for enzymatic saccharification. In that case, both the lignin in the solid phase and water-soluble lignin degradation products in the liquid phase would be present during enzymatic saccharification, and both could potentially serve as reductants in LPMO-catalyzed reactions. However, an alternative approach is to separate the solid and the liquid phases after pretreatment, and in that case only the solid phase would be present during enzymatic saccharification. Then, only the lignin in the solid phase would serve as reductant in LPMO-catalyzed reactions. The question then arises how the electron donor capacity of the pretreated biomass is divided between the solid and the liquid phases.

In this investigation we address the question about how the electron donor capacity is divided between the phases using pretreated biomass from a demonstration plant operated by a commercial company and a novel experimental set-up allowing controlled gas addition to six parallel reaction mixtures. Thereby, better control of the redox environment was achieved, and statistical analysis of results from parallel reaction mixtures was made possible. Even if LPMO is present in modern enzyme preparations, it is not always clear that its capacity is fully exploited due to insufficient supply of reductant and/or molecular oxygen. This makes an experimental set-up with gas addition advantageous, permitting controlled addition of oxygen. Research in this area provides more knowledge about LPMO-catalyzed reactions and guidance on how to best perform enzymatic saccharification in industrially relevant settings.

## 2 Materials and methods

### 2.1 Raw material and pretreatment

Chipped unbarked Norway spruce (*Picea abies*) was pretreated in the Biorefinery Demonstration Plant (BDP) (Örnsköldsvik, Sweden) by SEKAB E-Technology AB. The wood chips were impregnated with sulfur dioxide (approx. 2% based on the dry weight of the biomass), and subsequent pretreatment was performed using continuous steam explosion at 205°C for approx. 10 min. The pH of the generated slurry was approx. 1.8, and the dry-matter content was around 30%.

Prior to enzymatic saccharification experiments, the pH of the slurry was adjusted to 5.2 using a 10 M aqueous solution of sodium hydroxide, and the total solid (TS) content was adjusted to 12.5% (w/w) by dilution with ultra-pure water. The solid and liquid phases of the slurry were separated by centrifugation (Avanti J-26 XP, Beckman Coulter, United States). The solid phase was extensively washed with ultra-pure water until glucose was no longer detectable in the filtrate through analysis with a glucometer (Accu-Chek Aviva, Roche Diagnostics GmBH). The solids were then air-dried before further use. The dry-matter content of the washed and dried solids was 96.6% according to measurements using an HG63 moisture analyzer (Mettler-Toledo, Greifensee, Switzerland). Particles remaining in the liquid phase after centrifugation were removed by passing it through Nalgene™ Rapid-Flow™ sterile filters (0.2 µm, PES, Thermo Fisher Scientific, Waltham, MA, United States). The pretreated solids (PS) and the pretreatment liquid (PL) were then used in enzymatic saccharification experiments.

### 2.2 Enzymatic saccharification

Two series of enzymatic saccharification experiments were performed. One consisted of pretreatment liquid (PL) with microcrystalline cellulose (Avicel^®^ PH-101 obtained from Sigma-Aldrich, St. Louis, MO, United States) and the other consisted of pretreated solids (PS) with 20 mM sodium acetate buffer, pH 5.2. Reaction mixtures consisted of 8.75 g (dry-weight) solids (PS or Avicel, used as substrate in the enzymatic reaction), 61.25 g liquid phase (buffer or PL), 540 µL Cellic^®^ CTec3 (Novozymes A/S, Bagsvaerd, Denmark) (1.13 g/mL), and 88 µL antifoaming agent (Tween-80). A separate experiment was performed to assure that inclusion of antifoaming agent did not affect the enzymatic reaction (data not shown).

Enzymatic saccharification experiments were carried out using 125 mL gas wash bottles (Quickfit^®^ Drechsel bottle, Sigma-Aldrich). Aerobic and anaerobic conditions were achieved by continuous supply of air or N_2_ to the gas wash bottles through Polyamide 12 tubing (outer diameter 6 mm). The set-up could accommodate up to six gas wash bottles in parallel in each experiment, allowing for two sets of triplicates (one set with air and one set with N_2_) and subsequent statistical analysis of the results. For each gas supply, a gas distributor divided the gas into three strands, and each gas strand was connected with a flow meter (Porter F65, Parker, United States). The purity of the N_2_ was 99.996%. The gas flow was kept at one vvm (based on flow rate and reaction mixture volume). The gas wash bottles were positioned on a multipoint stirrer (Cimarec from Thermo Scientific) immersed in a water bath to allow for temperature control. The bottles were equipped with magnetic stirrer bars (cylindrical 40 × 8 mm), and the stirring speed was 215 rpm. Incubation was performed at 45°C for 72 h, and samples (0.5 mL each) were withdrawn after 24 h, 48 h, and 72 h by using an automatic pipette. Zero samples (0 h) were collected after adding enzyme. Reactions were conducted in triplicates, and the monosaccharides produced in the reactions were analyzed using HPAEC (Section 2.3.1).

After enzymatic saccharification experiments, the mixtures were centrifuged to separate the solid and the liquid phases. Solid phases were washed with abundant ultra-pure water until no glucose was detectable in the filtrate. Washed solids were air-dried until the dry-matter content was over 90%, and they were then sieved using sieve shakers (Retsch AS 200) with an aperture size of 100 μm to homogenize the solid fraction. Liquid phases were stored at 4°C until further analysis.

### 2.3 Analysis of liquid phase

#### 2.3.1 Analysis of monosaccharides and glucan conversion

Analysis of monosaccharides was performed using high-performance anion-exchange chromatography (HPAEC) with pulsed amperometric detection (PAD). The separation system consisted of an ICS-5000 system equipped with an electrochemical detector, a CarboPac PA1 (4 mm × 250 mm) separation column, and a CarboPac PA1 (4 mm × 50 mm) guard column (all from Dionex, Sunnyvale, CA, United States). The temperature of the column oven was kept at 30°C and all samples were diluted with ultra-pure water and filtered through 0.20 µm nylon membrane filters (Merck Millipore Ltd., Cork, Ireland). Samples were eluted for 25 min with ultra-pure water at a flow rate of 1 mL/min. The column was regenerated by washing for 11 min with a mixture containing 60% of an aqueous solution of 300 mM sodium hydroxide and 40% of an aqueous solution consisting of a mixture of 200 mM sodium hydroxide and 170 mM sodium acetate, followed by 3 min equilibration with ultra-pure water. External calibration standards of monosaccharide mixtures in the range of 0.5 mg/L–30 mg/L were prepared, and each sample was analyzed in triplicate. Data analysis was performed using the Chromeleon 7.1 software (Dionex).

The conversion of cellulose to glucose (glucan conversion) in the enzymatic hydrolysis was calculated by the ratio of glucose produced during enzymatic hydrolysis in relation to the glucan in the substrate using Eq. 1 (Lu et al., 2012)

$$Glucan\;conversion\;\left(\%\right)=\frac{produced\;glucose\;concentration\times M\times 0.9}{glucan\;content\times m}\times 100$$ (1)

In Eq. 1, the “produced glucose concentration” refers to the glucose concentration determined using HPAEC in (g/L) with potential glucose in the reaction mixture prior to the enzymatic reaction deducted. “M” refers to the total mass (in g) of the enzymatic reaction mixture (solid and liquid) in the beginning of the reaction, and “m” refers to the mass (in g) of total solids. The glucan content of PS was calculated on basis of compositional analysis (Section 2.4.1) and the glucan content of the model substrate Avicel PH-101 was assumed to be 97.6% on basis of literature data (Yu et al., 2012).

#### 2.3.2 Analysis of total phenolics

Folin - Ciocalteu’s reagent (Singleton et al., 1999) was used for determination of total phenolics in the PL. Vanillin was used as the calibration standard. The color generated after 40 min incubation at room temperature was measured as the absorbance at 760 nm using a BioTek Epoch Microplate Spectrophotometer (Agilent, Santa Clara, CA, United States). Reactions were performed in triplicates.

#### 2.3.3 Analysis of total carboxylic acid content

The total carboxylic acid content (TCAC) was determined as previously described (Wang et al., 2018a). Briefly, titration using an aqueous sodium hydroxide solution (200 mM) was performed in the range from pH 2.8 to pH 7.0.

#### 2.3.4 Analysis of total aromatic content

Total aromatic content (TAC) covers both aromatics (phenolic and non-phenolic aromatics) and heteroaromatics, such as 5-hydroxymethylfurfural (HMF) and furfural (Wang et al., 2018a). TAC was measured as absorbance units at 280 nm (AU_280_) using a UV 1800 spectrophotometer (Shimadzu, Kyoto, Japan) with a dilution factor of 1,000.

#### 2.3.5 Analysis of furan aldehydes

Quantitation of HMF and furfural was performed using a Thermo Scientific UltiMate 3,000 HPLC system (Dionex Softron GmbH, Germany) equipped with a detector. The eluents were ultra-pure water with 0.1% (v/v) formic acid (Eluent A) and acetonitrile with 0.1% (v/v) formic acid (Eluent B). The flow rate was 0.5 mL/min. Chromatographic separation was conducted on a Zorbax RRHT SB-C18 column (3.0 mm × 50 mm, 1.8 µm particle size) with 3% of eluent B for 3 min. This was followed by a 4 min cleaning step with 20% of eluent B, and finally the column was equilibrated for 4 min with 3% of eluent B. The absorption at 282 nm was recorded, and the temperature of the column oven was 40°C. An external calibration curve covering the interval 5 μM–250 µM and the Chromeleon 7.1 software were used for quantitation.

#### 2.3.6 Analysis of LPMO oxidation products

Tentative C1 and C4 oxidation products from LPMO-catalyzed reactions were analyzed using the ICS-5000 system and pulsed amperometric detection. Separation was performed on a CarboPac PA1 column for 20 min with a flow rate of 1 mL/min. Gradient elution was carried out for 1.5 min using an aqueous solution of 0.3 M sodium hydroxide, followed by elution for 15.5 min with an aqueous solution consisting of a mixture of 0.3 M sodium hydroxide and 0.5 M sodium acetate. Finally, an aqueous solution of 0.3 M sodium hydroxide was added for 3 min to condition the column before the next sample injection.

### 2.4 Analysis of pretreated solids

#### 2.4.1 Two-step treatment with sulfuric acid

The contents of carbohydrates and lignin (Klason lignin and acid-soluble lignin) of the solid were determined using the TSSA method. The analysis was performed according to the NREL/TP-510–42618 protocol (Sluiter et al., 2012), with some modifications. Monosaccharides were analyzed by using HPAEC instead of HPLC. Prior to monosaccharides analysis, samples were diluted with ultra-pure water and filtered through 0.20 µm nylon membranes (Section 2.3.1). Acid-insoluble lignin (Klason lignin) was determined gravimetrically by using glass crucibles with integral glass sintered discs (Pyrex 2, porosity 10 µm–16 µm). Acid-soluble lignin (ASL) was determined spectrophotometrically at λ 240 nm (UV-1800 spectrometer, Shimadzu, Kyoto, Japan). All analyses were performed in triplicates.

#### 2.4.2 Pyrolysis-gas chromatography/mass spectrometry

*Py*-GC/MS was used to determine the lignin-carbohydrate fraction of pretreated solids. The analysis was performed at the Biopolymer Analytical Platform (BAP) of the KBC Chemical-Biological Center (Umeå, Sweden). The instrument consisted of an oven pyrolyzer equipped with an autosampler (PY-2020iD and AS-1020E, Frontier Labs, Koriyama, Japan) connected to a GC/MS system (Agilent 7890A/5975C). The method has been described in detail by Gerber et al. (2016).

#### 2.4.3 Phenolic content of solids

The determination of the phenolic groups content of pretreated solids was performed by MoRe Research AB (Örnsköldsvik, Sweden) using a method based on the study by Lai et al. (Lai et al., 1990). The relative standard error was estimated to 10%**.**

#### 2.4.4 Fourier transform infrared spectroscopy

FTIR analysis was conducted at the Vibrational Spectroscopy Core Facility (ViSp) of the KBC Chemical-Biological Center. The samples were prepared using potassium bromide (Spectrograde KBr, Fisher Scientific, Waltham, MA, United States). The spectra were obtained on a Bruker IFS 66v/S FTIR spectrometer, equipped with a standard deuterated triglycine sulfate detector and fitted with a diffuse reflectance accessory (Bruker Corporation, Billerica, MA, United States). Spectra between 800 and 1800 cm^−1^ were recorded using 256 scans and 4 cm^−1^ resolution.

#### 2.4.5 2D-HSQC NMR

A portion (25 mg) of each sample was transferred to a 5 mm NMR-tube to which 600 μL DMSO-d6 was added. Samples were analyzed using 2D ^1^H-^13^C Heteronuclear Single Quantum Coherence (HSQC) NMR with a Bruker 600 MHz Avance III HD spectrometer equipped with a BBO cryoprobe and using the pulse program hsqcetgpsisp 2.2. For each of the 256 t1-increments, 32 scans were recorded with an inter-scan delay of 1.5 s, resulting in an experimental time of approximately 3 h and 50 min. Experiments were performed at 298 K. Alcohol-insoluble residue (AIR) of wood of Norway spruce was dissolved in DMSO-d6 and used as non-pretreated reference. Spectra were processed using Topspin 3.6 (Bruker Biospin, Germany) with Gaussian window function in F2 and a 90-degree shifted squared sine-bell window function in F1.

#### 2.4.6 Scanning electron microscopy

After washing with water and drying (Section 2.1), dried PS were dispersed onto carbon conductive tape mounted on an aluminum specimen stub and coated with 10 nm platinum using a carbon-sputter coater (Quorum Q150T-ES). The morphology of the PS samples was analyzed by field-emission scanning electron microscopy (FESEM, Carl Zeiss Merlin GmbH) using an in-lens secondary electron detector at a voltage of 3 kV and a probe current of 30 pA. The SEM analysis was conducted at the Umeå Core Facility Electron Microscopy (UCEM) of the KBC Chemical-Biological Center.

## 3 Results and discussion

### 3.1 Analysis of pretreated biomass

The liquid and solid fractions of pretreated softwood from the demonstration plant were analyzed to get an overview of lignin-related material and carbohydrates in the fractions. According to compositional analysis using TSSA (Table 1), the pretreated solids contained (DW) 44.5% glucan and 51.6% total lignin (Klason lignin and ASL). No other carbohydrates than glucan were detected. This indicates that the pretreatment conditions had been very harsh, as all hemicellulosic carbohydrates had been removed and as the lignin content was higher than the glucan content. From the beginning, it is the other way around, as untreated spruce wood contains 27.4% lignin and 41.7% cellulose (Sjöström, 1993). The aim with the pretreatment is to achieve an almost quantitative removal of hemicelluloses, without degrading cellulose, which is left for enzymatic saccharification. The fact that the total lignin content was higher than the glucan content indicates partial degradation of cellulose, formation of pseudo-lignin from hemicelluloses, or occurrence of both these events. Pseudo-lignin is a Klason-lignin-positive aromatic substance formed from carbohydrates during thermal treatment under harsh conditions (Shinde et al., 2018; Martín et al., 2022; Yao et al., 2022).

**TABLE 1:** Analysis of the solid phase of pretreated Norway spruce.

Analysis and constituent		PS^g^	PS-Air^g^	PS-N_2_ ^g^
TSSA^a^	Glucan	44.5 (1.6)	28.8 (1.2)	37.5 (1.0)
	Xylan	ND	ND	ND
	Mannan	ND	ND	ND
	Arabinan	ND	ND	ND
	Galactan	ND	ND	ND
	Klason lignin	44.8 (0.7)	56.2 (2.7)	47.9 (1.4)
	ASL^f^	6.8 (0.1)	6.3 (0.2)	5.8 (0.1)
	Ash	≤ 0.3	≤ 0.3	≤ 0.3
Py-GC/MS^b^	Carbohydrates	67.4 (1.0)	58.6 (1.0)	63.7 (1.1)
	G	21.3 (0.6)	28.7 (0.7)	24.7 (0.6)
	H	2.5 (0.1)	3.3 (0.1)	2.9 (0.1)
	Total lignin	24.2 (0.6)	32.6 (0.8)	28.0 (0.7)
	Carb./lignin	2.8 (0.1)	1.8 (0.1)	2.3 (0.1)
Phenolic hydroxyl groups^c^	mmol/kg DS^d^	760	1,030	970
	mmol/kg total lignin^e^	1,460	1,650	1810

^a^ Two-step treatment with sulfuric acid. Values given in percent dry weight with standard deviations in parentheses.

^b^ Values given in percent of peak area, according to method described by Gerber et al. (2016).

G, guaiacyl; H, *p*-hydroxyphenyl. Standard deviations in parentheses.

^c^ Method described by Lai et al. (1990), with relative standard error estimated to 10%.

^d^ Values given in mmol/kg dry solids.

^e^ Mmol/kg total lignin (compositional analysis).

^f^ ASL: acid-soluble lignin.

^g^ PS, pretreated solids; PS-Air, solid fraction after 72 h enzymatic saccharification with air; PS-N_2_, solid fraction after 72 h enzymatic saccharification with N_2_.

Analysis using *Py*-GC/MS (Table 1) suggested that the pretreated material predominantly (∼70%) consisted of carbohydrates and that around a quarter of the content was lignin. As expected for softwood (Ralph et al., 2004) such as spruce, the lignin predominantly consisted of G units (Table 1). With respect to the determined fractions, the discrepancy compared to the results from the TSSA analysis can be explained by pseudo-lignin being characterized as carbohydrates in the *Py*-GC/MS analysis, which has been observed previously (Wang et al., 2018a). Thus, compositional analysis using TSSA and *Py*-GC/MS indicated that the hemicelluloses had been quantitatively removed or degraded and that the pretreated material predominantly consisted of three substances: glucan, lignin, and pseudo-lignin.

**FIGURE 1:**
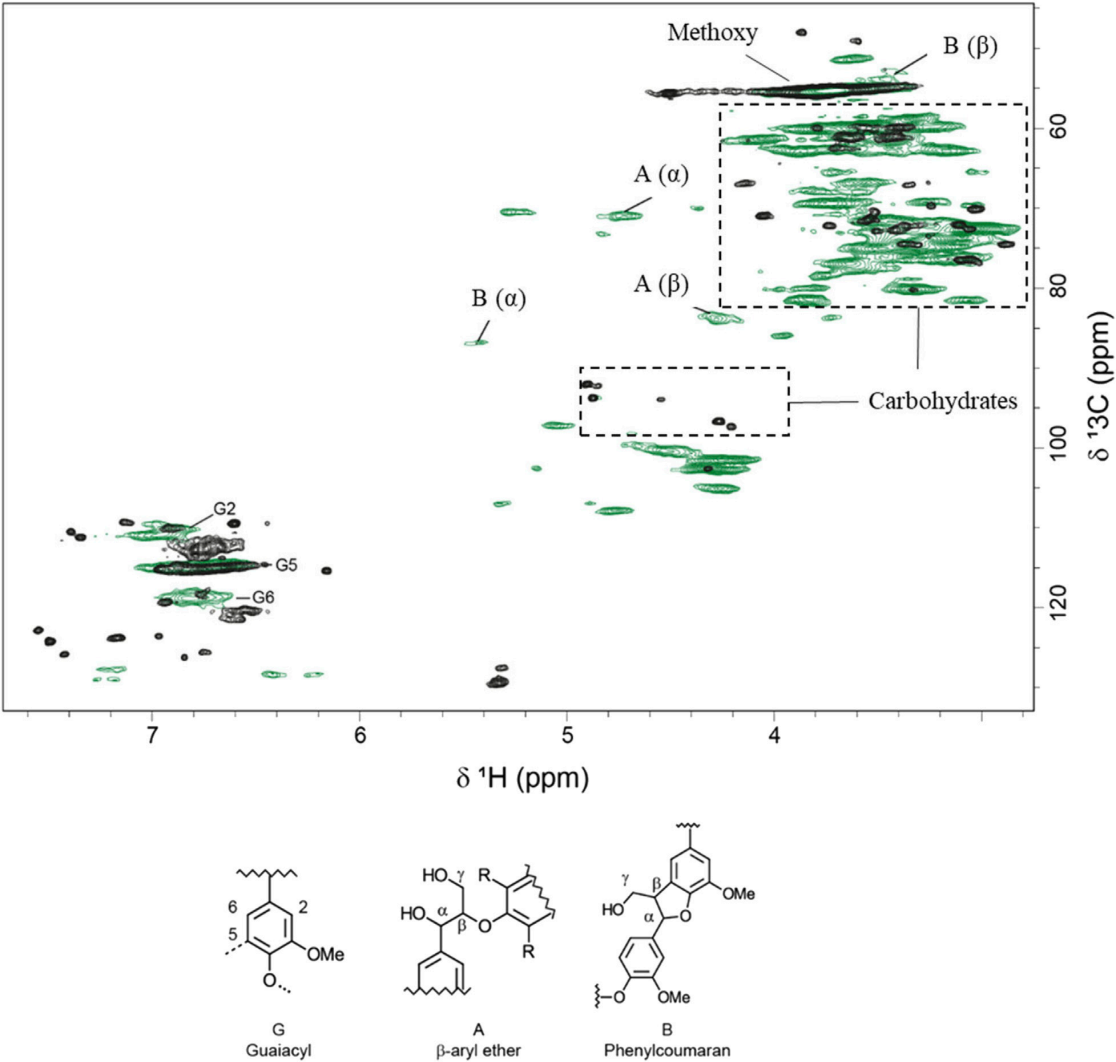
Overlay of two-dimensional ^1^H–^13^C HSQC NMR spectra of alcohol-insoluble residue from Norway spruce (in green) and pretreated solids (in black).

The pretreated solids were analyzed further using HSQC NMR (Figure 1). Pretreated solids exhibited relatively poor solubility in DMSO compared to non-pretreated Norway spruce wood, and the signals were weaker. The pretreatment affected the aromatic region of the lignin, as indicated by the lower intensity of G2 and G6 after pretreatment. Signals from common interunit lignin linkages, such as β-O-4 and phenylcoumaran, were weak in the pretreated material (Figure 1). These changes are in agreement with previous studies of lignin after pretreatment under acidic conditions (Yao et al., 2022). An attempt to solubilize more of the pretreated material using a mixture of DMSO-d6 and pyridine-d5 (Kim and Ralph, 2010) to obtain stronger signals was unsuccessful.

**FIGURE 2:**
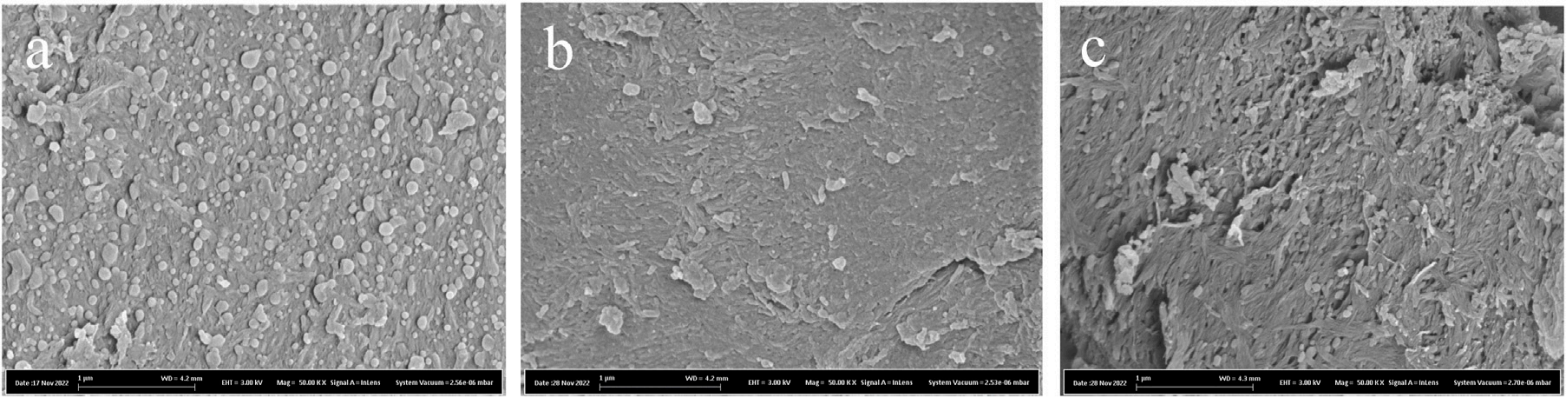
SEM micrographs of washed pretreated solids prior to enzymatic saccharification reactions **(A)**, and after 72 h of enzymatic saccharification in reactions supplemented with **(B)** air and **(C)** N_2_. The scale bar length is 1 µm.

SEM was used to further investigate the structure of the solid fractions. Micrographs of PS showed nano-sized particles or droplets on fibrillar surfaces (Figure 2A). There are previous reports on droplets on biomass surfaces caused by relocalization of lignin (Selig et al., 2007; Donohoe et al., 2008) and by pseudo-lignin formation (Sannigrahi et al., 2011) during pretreatment under acidic conditions. Such droplets could vary significantly in size, such as from 5 nm up to 10 μm (Donohoe et al., 2008) or 0.3 μm–8.0 μm (Sannigrahi et al., 2011). It is a possibility that harsh, acidic pretreatment conditions contributed to the formation of these structures (Figure 2A). After enzymatic saccharification with air, the surfaces appeared somewhat smoother (Figure 2B). This was less pronounced in reactions with N_2_ (Figure 2C), where the enzymatic saccharification had reached less far.

As expected considering the relatively high content of galactoglucomannan in spruce wood (Sjöström, 1993), mannose was the predominant hemicellulosic monosaccharide in the PL (Table 2). The total concentration of hemicellulosic sugars (mannose, xylose, galactose, and arabinose) (Table 2) was 36 g/L, and the concentration of glucose was around 31 g/L (Table 3). Even if it is not intentional to degrade cellulose in the pretreatment, the glucose could come either from hemicelluloses or from partial degradation of cellulose.

**TABLE 2:** Analysis of pH-adjusted pretreatment liquid and the liquid phases after 72 h saccharification.^a^

Property/substance	PL	PL-air	PL-N_2_
pH	5.1	4.6	5.0
TCAC (mM)^b^	192 (5)	261 (2)	244 (8)
Total phenolics (g/L)^c^	5.1 (0.3)	5.6 (0.2)	5.9 (0.6)
HMF (mM)	26.6 (0.2)	31.1 (0.3)	29.8 (2.1)
Furfural (mM)	32.2 (0.2)	0.5 (0.1)	0.6 (0.1)
TAC^d^	1.2 (0.1)	0.8 (0.1)	0.8 (0.1)
Mannose (g/L)^e^	20.0 (0.7)	25.0 (0.7)	26.8 (1.4)
Xylose (g/L)^e^	10.2 (0.4)	14.2 (0.3)	14.8 (0.7)
Galactose (g/L)^e^	3.9 (0.2)	5.0 (0.2)	5.3 (0.3)
Arabinose (g/L)^e^	2.3 (0.1)	2.7 (0.1)	2.9 (0.2)

^a^ Standard deviations are shown in parentheses.

^b^ Total Carboxylic Acid Content determined using titration with a solution of sodium hydroxide.

^c^ Concentration of total phenolics using the Folin-Ciocalteu assay with vanillin as the standard.

^d^ Total Aromatic Content indicated as absorbance units at 280 nm (AU_280_) with a dilution factor of 1,000.

^e^ Concentration of hemicellulosic monosaccharides. Values from PL, are zero samples while values from PL-Air and PL-N_2_, are from 72 h of saccharification.

**TABLE 3:** Sugar yields from reaction mixtures with pretreated solids or pretreatment liquid.^a^ ^,^ ^b^

Reaction Mixture		Glucose concentration (g/L)^c^				Glucan conversion (% w/w) after 72 h^d^	Increase in glucan conversion in aerated reaction after 72 h (%)
		0 h	24 h	48 h	72 h		
PS with buffer	Air	3.0 (0.4)	27.7 (2.6)***	38.2 (4.9)*	56.3 (5.2)***	85.9 (8.1)***	25
	N_2_	2.8 (0.4)	23.7 (2.1)	33.5 (3.5)	45.3 (4.1)	68.6 (6.3)	
Avicel with PL	Air	31.3 (1.2)	63.9 (1.1) ***	77.9 (2.5)***	85.7 (2.6)***	40.1 (1.5)***	14
	N_2_	30.6 (1.2)	52.4 (1.4)	65.8 (2.6)	78.4 (3.8)	35.2 (3.2)	

^a^ Enzymatic saccharification of pretreated solids (PS) in 20 mM sodium acetate buffer (pH 5.2) and of Avicel PH-101, in pretreatment liquid (PL).

^b^ Standard deviations in parentheses. Asterisks indicate significant differences (Student *t*-test) between aerobic and corresponding anaerobic conditions: ***p ≤ 0.01; ** 0.01 < *p* ≤ 0.05; * 0.05 < *p* ≤ 0.1.

^c^ Glucose concentrations (g/L) analyzed using HPAEC.

^d^ Glucan conversion (% w/w) was calculated according to Eq. 1, with a glucan content of 44.5% in PS, and 97.6% in Avicel PH-101.

The concentrations of substances in the pretreatment liquid (Tables 2, 3) can be compared to those found in a series of pretreatment liquids from Norway spruce pretreated with sulfur dioxide under low, medium, and high severity (Wang et al., 2018a; Martín et al., 2018). The TCAC content was just below 200 mM (Table 2), which is very high for a TS content of 12.5% considering that it was 203 mM after the most severe treatment when the TS was 25% (Wang et al., 2018a). The content of total phenolics was around 5 g/L, which again is very high compared to 1.6 g/L after the most severe treatment in a previous study of material with TS ∼30% (Martín et al., 2018). The combined concentration of furfural and HMF was around 59 mM (Table 2). Considering the TS, 12.5%, that is higher than the highest combined value for furfural and HMF reported by Martín et al. (2018), which was 87 mM at a TS of around 30%. The TAC value (1.2 with 1,000 × dilution) was also relatively high compared to previous studies, as Wang et al. (2018a) reported values from 0.59 up to 2.74 with TS 25% and 500 × dilution. That is consistent with the high values for total phenolics and furan aldehydes, as TAC covers both of these groups of substances.

The results of the analysis of the liquid phase are consistent with the results of the analysis of the solid phase, as both data sets point to very harsh pretreatment and, consequently, high contents of Klason lignin and phenolics. The content of lignin in the solid phase and the content of phenolics in the liquid phase would be relevant, as it may affect the ability of these fractions to serve as reductants in reactions with LPMO. In addition to that, substances in the PL might inhibit cellulolytic enzymes. Such substances include both sugars (causing feedback inhibition of cellulases) and aromatics, such as phenolics (Zhai et al., 2016; Martín et al., 2022).

The total monosaccharide concentration (∼67 g/L) of the PL used in this work was similar to the total monosaccharide concentrations (64 g/L–77 g/L) of the PLs studied by Wang et al. (2018a). As all PLs studied by Wang et al. (2018a) had total monosaccharide concentrations that were inhibitory to the cellulolytic enzyme preparations, it is safe to assume that the PL used in this work also caused sugar inhibition. All PLs studied by Wang et al. (2018a) were also clearly inhibitory to cellulolytic enzyme preparations with respect to other substances than monosaccharides, such as phenolics, despite that the concentrations of phenolics in the PLs [0.7 g/L–1.6 g/L according to data from Martín et al. (2018)] were much lower than in the PL used in this work (∼5 g/L). It is therefore likely that the PL used in this work also contained inhibitory concentrations of phenolics.

### 3.2 Sugar yields in enzymatic saccharification experiments

The enzymatic saccharification reactions contained the same amount of solids (8.75 g Avicel or PS), but different glucan content. Whereas Avicel consists almost exclusively of glucan (in the form of microcrystalline cellulose), the PS had a glucan content of 44.5%, and therefore the reaction mixtures with PS contained 3.89 g glucan rather than 8.75. Reactions with PS also contained lignin and pseudo-lignin, which have a negative effect on saccharification (Shinde et al., 2018; Martín et al., 2022; Yao et al., 2022). Avicel would therefore be a much better substrate than PS, but the reactions with Avicel also contained PL and therefore also sugars and phenolics that inhibit enzymatic saccharification. For these reasons, it was not self-evident which set of reactions that would result in the highest enzymatic saccharification yields.

Data on glucose concentrations and glucan conversion are shown in Table 3. As expected (Arantes and Saddler, 2011), the glucose concentrations increased rapidly in the beginning and leveled off at the end. The glucose concentrations (in g/L) generated between the zero sample and 72 h were: PS/air, 53.3; PS/N_2_ 42.5; Avicel/air, 54.4; Avicel/N_2_, 47.8. Thus, reactions with air always resulted in more glucose than the corresponding reactions with N_2_ (25% more for PS and 14% more for Avicel). This can be attributed to the catalytic action of LPMO, and it was apparent both with PS and PL without any addition of external reductant. Although the glucose yields of enzymatic saccharification were rather similar for reactions with air (53 g/L–54 g/L) and for reactions with N_2_ (42 g/L–48 g/L), the glucan conversion was higher for reactions with PS (68.6%–85.9%) than for reactions with Avicel (35.2%–40.1%). This phenomenon can be attributed to the presence of enzyme inhibitors in PL having a negative effect on the reactions with Avicel, and perhaps also to the enzyme:glucan ratio being more than twice as high in experiments with PS as in experiments with Avicel.

This work differs from previous studies of LPMO-catalyzed reactions with pretreated biomass in several different ways; as it (i) covers the separated solid and liquid phases from the pretreated biomass, (ii) involves controlled continuous gas addition using air or N_2_, (iii) is based on hydrothermal pretreatment of spruce wood with sulfur dioxide in a demonstration plant, (iv) involves no redox-active chemicals except oxygen in air in additions after pretreatment, and (v) involves detailed characterization of the liquid and solid phases, for instance with respect to the occurrence of pseudo-lignin. In a series of experiments with steam-pretreated lodgepole pine with different lignin content, Hu et al. (2014) found that addition of an external reducing agent (gallate) had a positive effect on the LPMO reaction only with a completely delignified substrate, but not when the lignin content was 12% or higher. Rodríguez-Zúñiga et al. (2015) investigated washed pretreated solids from sugarcane bagasse, wheat straw, and corn stover. The gas phase was not controlled, but determination of gluconic acid, an oxidation product from the LPMO reaction, suggested that substrates with low (9.6% or less) Klason lignin content were associated with no or very small LPMO activity. Müller et al. (2015) showed improved saccharification of pretreated birch wood in flasks containing air in the headspace compared to reactions in flasks in which the headspace was flushed with N_2_ and in which cysteine was added to the reaction mixtures to remove residual oxygen. Chylenski et al. (2017) investigated saccharification of sulfite-pulped softwood, and therefore their biomass contained much less lignin (4%–9% acid-insoluble lignin) than that used by Müller et al. (2015) or that used in the present study. As a result, improved saccharification with air in the headspace was observed only when an electron donor, such as ascorbic acid or lignosulfonates, was added to the reaction mixtures (Chylenski et al., 2017). These results agree with our findings, as the lignin content of the solid phase used in our experiments was clearly higher than the lignin contents of substrates for which no or very little LPMO activity has been detected without addition of an external reductant.

### 3.3 Analysis of liquid phase after enzymatic saccharification

**FIGURE 3:**
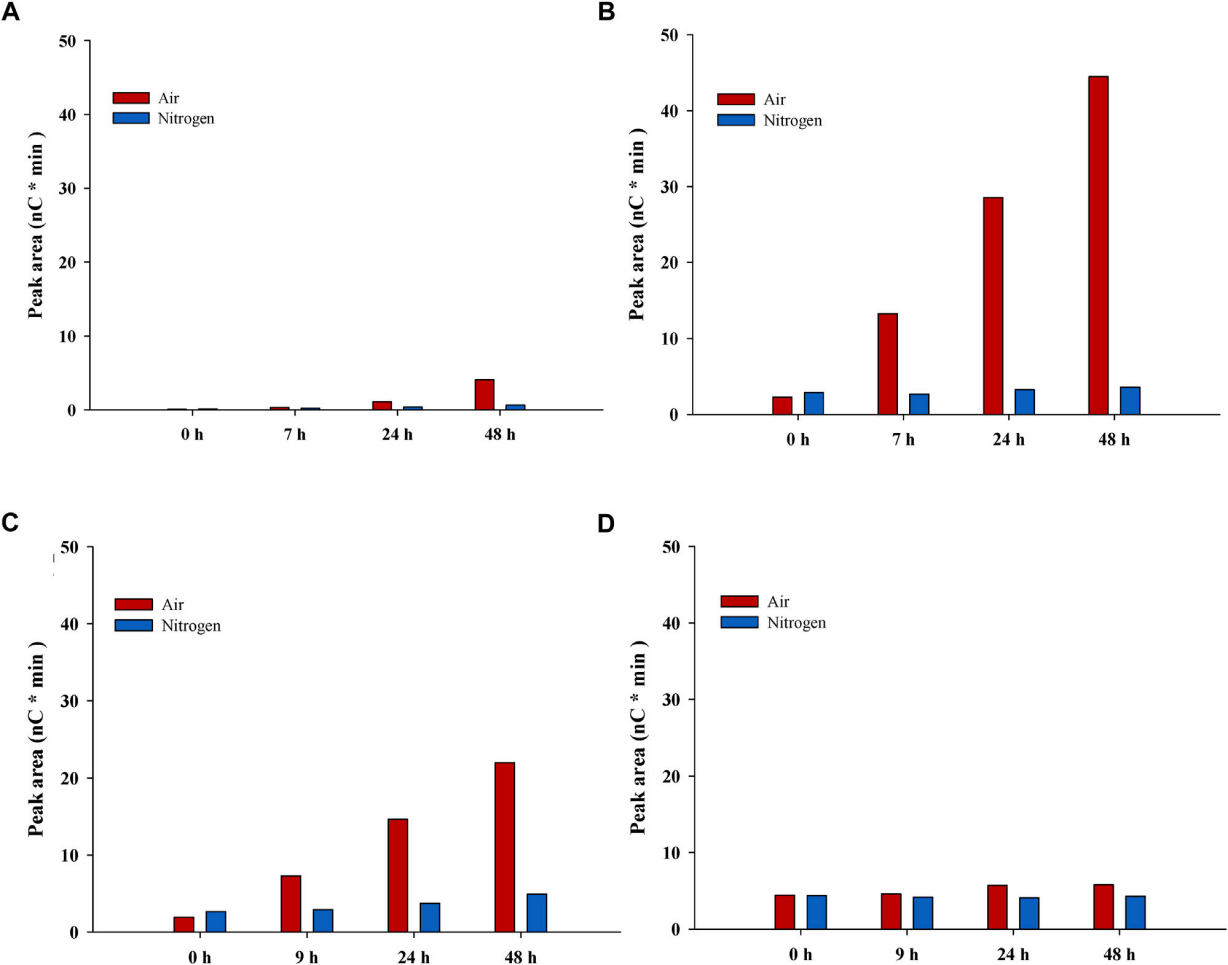
Estimation of oxidation products from LPMO-catalyzed reactions. The figure shows data for peaks with oxidation products from reactions with pretreated solids in buffer **(A)** and **(B)** and with Avicel in pretreatment liquid **(C)** and **(D)**. Left-hand panels **(A)** and **(C)** show the tentative C1 product, while right-hand panels **(B)** and **(D)** show the tentative C4 product. Red bars, reactions with air; blue bars, reactions with N_2_.

The liquid phases at the end of the reactions with Avicel (PL-Air and PL-N_2_) were analyzed with regard to other substances than glucose (Table 2; Figure 3). Smaller increases (0.4 g/L–6.8 g/L) in the concentrations of sugars other than glucose (Table 2) can be attributed to enzymatic saccharification of oligosaccharides and disaccharides, and also to concentration of reaction mixtures at the end of the experiments due to evaporation caused by gas flow.

The pH decreased somewhat in PL-Air, and the TCAC value was higher than for PL-N_2_. It is a possibility that oxidation products from LPMO-catalyzed reactions contribute to the high acid content in PL-Air. This is supported by data in Figure 3, which shows analysis of tentative C1 and C4 oxidation products. As judged from the elution time, the tentative C1 product [RT (retention time) 8.83] would correspond to gluconic acid (RT 8.84 for gluconic acid reference), and, at the end of the reaction (48 h), the peak area was much larger for reactions with air than for reactions with N_2_ (Figure 3C). The area of an unidentified peak at RT 13.4 increased for reactions with air, which was not observed for reactions with N_2_ (Figure 3B). This could tentatively reflect formation of a C4 oxidation product. Minor concentrations of the tentative C1 and C4 products were present also in reaction mixtures with N_2_, but that could be due to that those reactions were not completely devoid of molecular oxygen or other oxidants. Also, N_2_ contains molecular oxygen as contaminant, so small amounts of molecular oxygen would be added throughout the experiment even in reactions with N_2_.

LPMO-catalyzed cleavage of cellulose leads to oxidation of carbons in the β-1,4-glycosidic bonds, i.e., oxidation of C1 or C4. Using Cellic CTec2, Cannella et al. (2012) studied C1 oxidation products, such as gluconic acid and cellobionic acid, in reactions with pretreated wheat straw. Müller et al. (2015) quantified C4-oxidized cellobiose in reaction mixtures with pretreated birch wood and Cellic CTec2.

With regard to aromatic and heteroaromatic substances, the concentrations of total phenolics and HMF did not change very much throughout the experiment (Table 2). The concentration of furfural decreased from 32 mM in PL to around 1 mM in PL-Air and PL-N_2_. This is because furfural is much more volatile than HMF and the gas flow will remove it, as has also been observed in previous studies (Larsson et al., 1999). The TAC value decreased somewhat in both PL-Air and PL-N_2_, which can be attributed to the removal of furfural, as both aromatics and heteroaromatics contribute to TAC. The total phenolic content was somewhat higher after the reactions than in the original PL (Table 2). One reason for this could be contribution to total phenolics by the enzyme preparation, which was not yet added to the PL when samples for analysis of total phenolics were withdrawn.

### 3.4 Analysis of solid phase after enzymatic saccharification

As expected on basis of the saccharification data in Table 3, the glucan (TSSA) and carbohydrate (Py-GC/MS) contents of PS-Air and PS-N_2_ decreased compared to that of PS, while Klason lignin (TSSA) and total lignin (Py-GC/MS) increased (Table 1). The changes compared to PS were consistently larger for PS-Air than for PS-N_2_. This can also be seen as shifts in the carbohydrate:lignin ratio according to the Py-GC/MS analysis (Table 1).

Spruce lignin contains around 20.5 phenolic hydroxyl groups per 100 C9 (phenylpropane) units (Adler, 1977). The content of phenolic hydroxyl groups on dry solids increased in PS-Air and PS-N_2_ compared to PS (Table 1). This is expected, as the enzymatic saccharification of cellulose increases the fraction of lignin. When calculated as phenolic hydroxyl groups on total lignin (Klason lignin and ASL), the values for PS-Air and PS-N_2_ were still slightly higher than for PS.

**FIGURE 4:**
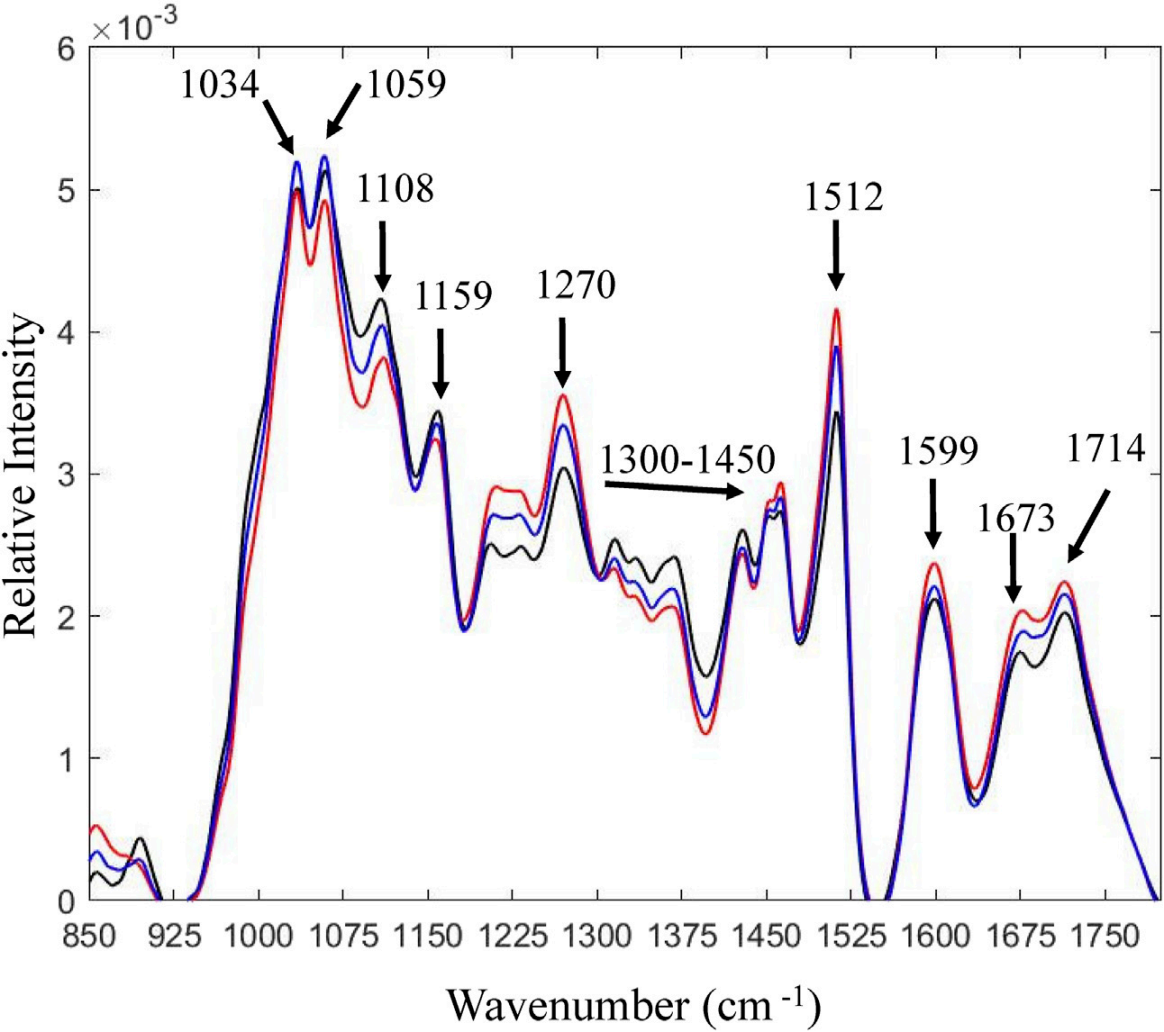
FTIR analysis of pretreated solids and solid fractions after 72 h of enzymatic saccharification. Black line, pretreated solids; blue line, reaction with N_2_; red line, reaction with air. Observed differences reflect relative compositional changes [total area normalization was performed on the spectra using MATLAB software developed by ViSp (Vibrational Spectroscopy Core Facility)].

Solid residues from PS-Air and PS-N_2_ reactions were analyzed using HSQC-NMR and FTIR. As for PS (Figure 1), the solubility was rather poor and the signals were relatively weak. Using FTIR, the solid fraction after enzymatic saccharification was compared to PS (Figure 4). Lower wavenumber regions, such as 1,030 cm^−1^–1,060 cm^−1^ and 1,108 cm^−1^ (ring vibrations) were assigned to polysaccharides (Labbé et al., 2005; Fackler et al., 2010; Durmaz et al., 2016). Lower band intensities for the reaction with air are consistent with more efficient enzymatic saccharification. This is also supported by differences observed for the bands at ∼ 1,150 cm^−1^ (Labbé et al., 2005) and 1,158 cm^−1^ (Pandey and Nagveni, 2007), which are associated with C-O-C asymmetric vibrations. Bands from 1,300 cm^−1^ to 1,450 cm^−1^ were assigned to C-H vibrations attributed to cellulose (Labbé et al., 2005). Lignin exhibits characteristic bands at 1,510 cm^−1^ and 1,600 cm^−1^. These bands are assigned to aromatic carbon-carbon double bonds (Sarkanen and Ludwig, 1971) and the band at 1,510 cm^−1^ is especially assigned for G units of lignin (Faix, 1991). In addition, an increase at 1,595 cm^−1^ is associated with aromatic skeletal vibrations (Faix, 1991; Faix and Böttcher, 1992) and indicates formation of pseudo-lignin (Wang et al., 2018b). According to previous studies (Faix, 1991; Pandey, 1999; Pandey and Nagveni, 2007), the band at 1,268 cm^−1^ is attributed to guaiacyl rings and carbonyl stretching in lignin. The band at 1,714 cm^−1^ is related to C = O stretching in unconjugated ketones, associated with pseudo-lignin (Sannigrahi et al., 2011), whereas the band at 1,672 cm^−1^ has been assigned to conjugated carbonyl groups (Gillgren et al., 2017).

## 4 Conclusion

The benefits of exploiting the LPMO present in a commercial enzyme mixture was investigated in experiments with softwood pretreated using continuous steam explosion with sulfur dioxide as catalyst. Harsh pretreatment conditions created a solid phase consisting mainly of Klason lignin and cellulose, with pseudo-lignin but without detectable amounts of hemicelluloses, and a liquid phase with high contents of lignin-derived phenols. Experiments with reaction mixtures that were continuously supplied with air or N_2_ indicate that both insoluble lignin in the solid phase and water-soluble lignin fragments in the liquid phase efficiently served as reductants in LPMO-supported saccharification of cellulose. Although relatively high concentrations of pretreatment by-products in the liquid phase had a suppressing effect on the saccharification reactions due to inhibition of cellulolytic enzymes, they also had a positive effect in the presence of air by supporting the LPMO reaction. Further research is needed to elucidate more details regarding the effects of water-soluble lignin fragments and potential effects of pseudo-lignin on LPMO-supported saccharification of cellulose.

## Data Availability

The original contributions presented in the study are included in the article/Supplementary Material, further inquiries can be directed to the corresponding author.
